# Prescribing antibiotics for children with dengue infection in Taiwan: who are at risk and who are high prescribers?

**DOI:** 10.1093/intqhc/mzae052

**Published:** 2024-06-15

**Authors:** Yi-Jung Shen, Chia-En Lien, Yiing-Jenq Chou, Theodore Tsai, Nicole Huang

**Affiliations:** Institute of Hospital and Health Care Administration, College of Medicine, National Yang Ming Chiao Tung University, Taipei 112, Taiwan; Department of Health Law, Policy and Management, Boston University School of Public Health, Boston, MA 02118, United States; Medigen Vaccine Biologics Corporation, Taipei 114, Taiwan; Institute of Public Health, College of Medicine, National Yang Ming Chiao Tung University, Taipei 112, Taiwan; Institute of Public Health, College of Medicine, National Yang Ming Chiao Tung University, Taipei 112, Taiwan; Office of the Deputy Superintendent, National Yang Ming Chiao Tung University Hospital, Yilan County 260, Taiwan; Takeda Vaccines, Cambridge, MA 02139, United States; Institute of Hospital and Health Care Administration, College of Medicine, National Yang Ming Chiao Tung University, Taipei 112, Taiwan

**Keywords:** antimicrobial resistance, antibiotic stewardship, dengue, Taiwan

## Abstract

Inappropriate antibiotic use contributes to antimicrobial resistance, a global public health threat. The non-specific manifestations of dengue, itself a growing public health threat, lead to avoidable empiric antibiotic prescription, particularly in children. In this national pooled population-based cross-sectional study, we evaluated child and physician characteristics associated with antibiotics prescription in confirmed dengue cases in Taiwan. Linking national health care insurance claims and reports of confirmed dengue cases from 2008 to 2015, there were 7086 children with confirmed dengue with 21 744 outpatient visits and 2520 inpatient admissions. We assessed the presence of antibiotic prescription in outpatient and inpatient settings separately a week before or after the confirmation date. Logistic regression models with generalized estimating equations were applied to identify patient, practitioner, and other factors associated with antibiotic prescription. A total of 29.4% of children <18 years old with dengue who did not have a concomitant bacterial infection were prescribed antibiotics during the 14-day assessment period. Antibiotics prescription was reduced from 13.5% to 6.3% and from 43.2% to 19.3% in outpatient and inpatient settings, respectively, after dengue was confirmed. Young children were more likely to receive antibiotics. Significant variations in antibiotic prescribing across physicians were observed only in outpatient settings: physicians ≥60 years old and physicians practicing at clinics and in non-urban facilities were more likely to prescribe antibiotics. Antibiotics were less likely to be prescribed during an exceptional 2-year epidemic than in other years. Antibiotic prescribing for dengue, an arboviral infection affecting half of the global population, was shown to occur in 29% of paediatric cases in Taiwan. That potentially avoidable antibiotic consumption could be reduced by improving antibiotic stewardship, informed by understanding the conditions under which antibiotics are prescribed and the availability of prevention strategies for viral diseases, including dengue. We identified a number of such factors in this national population-based study.

## Introduction

The widespread misuse of antimicrobial drugs is a leading cause of escalating rates of antibiotic resistance [1]. Antibiotics are commonly prescribed inappropriately to treat viral infections or when a given antibiotic is used but is not recommended [2]. Dengue, an acute arthropod-borne viral infection that is common in tropical and subtropical zones, is a growing global public health concern because of globalization and climate change. Dengue manifestations are non-specific or similar to the symptoms and signs of other febrile illnesses [3] that may be difficult to distinguish from infections that require antibiotic treatment, such as leptospirosis, particularly as point-of-care tests are not readily available. In such scenarios, inappropriate antibiotic prescription is unsurprising.

Children are at high risk of dengue in endemic areas, and in one study antibiotics were prescribed in over 50% of children with fever symptoms [4]. The decision to prescribe antibiotics is complex [5] and multifactorial. In addition to biological factors, child and parent characteristics and provider and environmental factors are involved in such decisions [6, 7]. Awareness of appropriate antibiotic use varies according to child characteristics and parental socio-economic factors [8–10]. Furthermore, substantial variation in antibiotic prescriptions among physicians has been observed [11].

Although substantial literature describes the inappropriate prescription of antibiotics for acute viral respiratory illnesses [12–15], few studies have assessed antibiotic prescription practices for children with dengue. Comprehensive analyses of the factors associated with inappropriate antibiotic prescription for paediatric dengue infection are lacking. Similar to many subtropical countries, periodic dengue outbreaks occur in Taiwan [16]. Taiwan has a well-established national infectious disease surveillance system and a National Health Insurance (NHI) database of claims data. When linked, they provide a unique opportunity for a national population-based evaluation of a wide array of factors potentially influencing antibiotic use in laboratory-confirmed dengue cases [17]. Our objective was to evaluate patient to physician characteristics associated with antibiotic prescription among children before and after dengue confirmation in outpatient and inpatient settings in a pooled population-based cross-sectional study. Taking advantage of the periodicity of dengue outbreaks in Taiwan, we also examined changes in prescription patterns during an unusual 2-year-long epidemic (2014 and 2015) compared with other years.

## Materials and methods

### Data source and study sample

Taiwan’s NHI scheme covers 99.9% of the island’s population, and all medical encounters must be reported to the National Health Insurance Research Database (NHIRD) for reimbursement. In this study, children with dengue infection were identified from the Notifiable Disease Dataset of confirmed cases, which is managed and maintained by the Taiwan Centers for Disease Control. The index date for cases was defined as the date of dengue confirmation. Point-of-care diagnostic tests for dengue were not available in Taiwan during the study period. Our study sample included children <18 years with confirmed dengue between 1 January 2008 and 31 December 2015. We kept only the first record if children had multiple records within 3 months. We obtained child, parental, and health care providers’ characteristics and medical care utilization data from the NHIRD. The validity and quality of the NHIRD have been assessed repeatedly [18, 19].

### Variables

The main dependent variable was the presence or absence of an antibiotic prescription within the 14-day assessment period (i.e. 7 days before and after the confirmation date). Antibiotic prescription records with concomitant bacterial infection diagnosis were excluded from the analyses. We used Anatomical Therapeutic Chemical (ATC) classification codes to identify the types of antibiotics prescribed (i.e. the first three digits of the J01 ATC codes). Topical antibiotic prescriptions were excluded from the analyses.

Children’s age, sex, health status, and parental socio-economic status (SES) were included in the analyses. A dichotomous health status variable was constructed as a dichotomous variable indicating whether the child had an illness listed on the Catastrophic Illness List designated by the NHI Administration. Parental SES was inferred by linking the patient’s identifier and birth date to NHI enrolment files and was defined on the basis of parents’ insurable wages. Among parents with a well-defined monthly wage, income was divided into <TWD30 000 and ≥TWD30 000 (TWD30 ≌ 1USD). A small number of parents without well-defined monthly wages were identified separately. We identified 2014 and 2015 as epidemic years and the remaining years as non-epidemic periods for analysis. The examined physician characteristics were age, sex, specialty, average monthly patient volume, and characteristics of their practice setting (including ownership and accreditation level). Physician’s age was divided into three groups: <35, 35–59, and ≥60 years. Specialty was divided into family medicine, internal medicine, otolaryngology, emergency medicine, paediatrics, and others. The average monthly patient volume was calculated on the basis of the number of outpatient visits or inpatient admissions 1 year before the index date. These volumes were divided into tertials. Ownership was divided into private and public. Accreditation level was divided into medical centres, regional and district hospitals, and clinics according to Taiwan’s medical facility accreditation regulations and procedures. Under the current accreditation regulations, medical centres are tertiary care hospitals with the highest quality of care standards.

### Statistical analyses

For patients with dengue, descriptive statistics were computed for the rates of antibiotic prescriptions and the patient-level and provider-level characteristics. Pre- and post-dengue confirmation prescription patterns in outpatient and inpatient settings were analysed separately. Owing to the hierarchical nature of the data, a generalized estimating equation was employed to control the probable clustering effects of medical care visits for each physician. The outcome of interest was the presence or absence of an antibiotic prescription in a medical encounter for dengue, for which we utilized a log-link function considering the dichotomous nature of the dependent variable. The exchangeable correlation structure was assumed, and all patient-level and provider-level characteristics were included in the models to estimate the adjusted odds ratios (ORs) and 95% confidence intervals (CIs). The index year was included as a covariate in the models. Sensitivity analyses were (i) visits with dengue diagnosis but without a concomitant bacterial diagnosis and (ii) all visits, including those with a concomitant bacterial diagnosis. The sensitivity analyses provided robust results. All analyses were conducted using SAS 9.4. All tests were two-tailed and *P*-value <0.05 was defined as statistical significance.

## Results

Children with incomplete patient-level information and no medical care visits within 7 days before and after the confirmation date were excluded (*N* = 361; 4.8%). The final study population included 7086 children with dengue infections. A total of 21 744 outpatient visits and 2520 inpatient admissions with the 14-day assessment period were included in the final analysis. Among 7086 children <18 years old with confirmed dengue between 2008 and 2015, 90.8% had dengue during the epidemic period (2014–15), 66% were >10 years, and only 2.1% of the patients had the existing major comorbidities (Table 1). Overall, 29.4% of confirmed dengue cases received antibiotic treatment during the 14-day assessment period. Among children who only had outpatient visits, 23.4% received a course of antibiotics. Among children who were hospitalized, 40.4% received antibiotics. In both the outpatient and inpatient settings, the most commonly prescribed antibiotics were first-generation cephalosporins. In all, 17.3%–26.3% and 10.0%–15.0% of the patients with dengue were prescribed both penicillin and macrolide antibiotics in the outpatient and inpatient settings, respectively (Table 2).

**Table 1.** Patient characteristics and medical utilization patterns among 7086 confirmed paediatric dengue patients, Taiwan, 2008–15.

	Child dengue patients (*N* = 7086)
	*N* (%)
Epidemic period	
No	652 (9.2)
Yes	6434 (90.8)
Patient characteristics	
Child age (years)	
<1	145 (2.0)
1–4	626 (8.8)
5–9	1642 (23.2)
10–14	2649 (37.4)
15–17	2024 (28.6)
Child sex	
Male	4091 (57.7)
Female	2995 (42.3)
Parental SES	
<TWD30 000	3115 (44.0)
≥TWD30 000	2155 (30.4)
Others	1816 (25.6)
Child major comorbid conditions	
No	6940 (97.9)
Yes	146 (2.1)
Medical utilization	
Any antibiotics prescribed	
No	5002 (70.6)
Yes	2084 (29.4)
Outpatient visit	
No	135 (1.9)
Yes	6951 (98.1)
Outpatient visit before confirmation	
No	1682 (23.7)
Yes	5404 (76.3)
Outpatient visit after confirmation	
No	1390 (19.6)
Yes	5696 (80.4)
Hospitalization	
No	4584 (64.7)
Yes	2502 (35.3)
Hospitalization before confirmation	
No	6467 (91.3)
Yes	619 (8.7)
Hospitalization after confirmation	
No	5192 (73.3)
Yes	1894 (26.7)

TWD30 ≌ 1USD.

**Table 2.** Top five antibiotics prescribed for paediatric dengue encounters.

	Outpatient setting						Inpatient setting					
	Pre-dengue confirmation			Post-dengue confirmation			Pre-dengue confirmation			Post-dengue confirmation		
Rank	Antibiotic	*N*	%	Antibiotic	*N*	%	Antibiotic	*N*	%	Antibiotic	*N*	%
1	Cefalexin	470	20.6	Cefalexin	246	19.4	Cefazolin	286	38.5	Cefazolin	352	37.2
2	Amoxicillin	393	17.3	Cefradine	200	15.8	Azithromycin	74	10.0	Azithromycin	104	11.0
3	Cefradine	372	16.3	Amoxicillin	190	15.0	Cefalexin	50	6.7	Cefalexin	92	9.7
4	Clindamycin	148	6.5	Amoxicillin and beta-lactamase inhibitor	83	6.6	Cefradine	50	6.7	Amoxicillin and beta-lactamase inhibitor	47	5.0
5	Cefaclor	116	5.1	Azithromycin	60	4.7	Ceftriaxone	34	4.6	Gentamicin	46	4.9

### Factors associated with antibiotic prescription

#### Antibiotic prescription in the outpatient setting

Antibiotic prescriptions were found for 9.7% of outpatient visits during the 14-day assessment period (Table 3). The proportion was lower for outpatient visits during the epidemic period (9.4%) than in other years (11.9%)—(OR: 0.74; 95% CI: 0.63–0.88; Table 4). The proportions of visits with antibiotic prescriptions were similar across children except for the age categories; the lowest proportion was for infants (4.5%), and the highest proportion was for children aged 15–17 years (11.3%). In contrast, major variations were observed across physician characteristics for those prescribing antibiotics. Physicians who were aged ≥60 years (OR: 1.78; 95% CI: 1.26–2.50), otolaryngologists (OR: 2.87; 95% CI: 2.22–3.70), and those whose practice facilities were located in non-urban areas (OR: 1.26; 95% CI: 1.07–1.50) were significantly more likely to prescribe antibiotics than other physicians. Regarding accreditation level, physicians who practiced at clinics had the highest likelihood of prescribing antibiotics to children with dengue (OR: 1.46; 95% CI: 1.04–2.06).

**Table 3.** Outpatient antibiotic use for confirmed dengue by patient and physician characteristics, in pre- and post-dengue diagnosis visits, Taiwan, 2008–15.

	Total		Pre-dengue confirmation			Post-dengue confirmation		
		Antibiotics use		Antibiotics use			Antibiotics use	
	*N* (%)	%	*N* (%)	%	*P*	*N* (%)	%	*P*
Total visits	21 744	9.7	10 005	13.5		11 739	6.3	
Epidemic period					.27			.12
No	1886 (8.7)	11.9	1159 (11.6)	14.6		727 (6.2)	7.7	
Yes	19 858 (91.3)	9.4	8846 (88.4)	13.4		11 012 (93.8)	6.3	
Patient characteristics								
Child age (years)					<.01			.31
<1	492 (2.3)	4.5	240 (2.4)	5.0		252 (2.1)	4.0	
1–4	1955 (9.0)	7.9	995 (9.9)	10.4		960 (8.2)	5.4	
5–9	5009 (23.0)	8.8	2591 (25.9)	10.7		2418 (20.6)	6.8	
10–14	8151 (37.5)	9.7	3678 (36.8)	13.7		4473 (38.1)	6.3	
15–17	6137 (28.2)	11.3	2501 (25.0)	18.4		3636 (31.0)	6.4	
Child sex					.47			.22
Male	12 594 (57.9)	9.6	5773 (57.7)	13.8		6821 (58.1)	6.1	
Female	9150 (42.1)	9.7	4232 (42.3)	13.3		4918 (41.9)	6.7	
Parental SES					.17			.32
<TWD30 000	9503 (43.7)	9.7	4385 (43.8)	13.3		5118 (43.6)	6.7	
≥TWD30 000	6717 (30.9)	9.4	3157 (31.6)	13.0		3560 (30.3)	6.2	
Others	5524 (25.4)	9.8	2463 (24.6)	14.7		3061 (26.1)	5.9	
Child major comorbid conditions					.57			.70
No	21 248 (97.7)	9.6	9754 (97.5)	13.5		11 494 (97.9)	6.3	
Yes	496 (2.3)	10.9	251 (2.5)	14.7		245 (2.1)	6.9	
Physician characteristics								
Physician age (years)					.01			<.01
<35	1932 (8.9)	6.2	657 (6.6)	11.1		1275 (10.9)	3.6	
36–59	17 631 (81.1)	9.6	8137 (81.3)	13.4		9494 (80.9)	6.4	
≥60	2181 (10.0)	13.0	1211 (12.1)	15.8		970 (8.3)	9.5	
Physician sex					.01			.25
Male	17 471 (80.3)	10.1	8568 (85.6)	13.9		8903 (75.8)	6.5	
Female	4273 (19.7)	7.7	1437 (14.4)	11.4		2836 (24.2)	5.9	
Physician specialty					<.01			<.01
Family medicine	2622 (12.1)	7.1	1420 (14.2)	10.1		1202 (10.2)	3.5	
Internal medicine	2659 (12.2)	7.5	1088 (10.9)	11.8		1571 (13.4)	4.5	
Otolaryngology	3506 (16.1)	17.9	2261 (22.6)	20.2		1245 (10.6)	13.6	
Emergency	3397 (15.6)	8.5	1183 (11.8)	14.5		2214 (18.9)	5.3	
Paediatrics	8704 (40.0)	7.8	3575 (35.7)	11.0		5129 (43.7)	5.7	
Others	856 (3.9)	13.6	478 (4.8)	13.0		378 (3.2)	14.3	
Average monthly volume					.25			<.01
Low	7176 (33.0)	6.9	1971 (19.7)	13.7		5205 (44.3)	4.3	
Medium	7175 (33.0)	10.2	3720 (37.2)	12.8		3455 (29.4)	7.3	
High	7393 (34.0)	11.9	4314 (43.1)	14.1		3079 (26.2)	8.7	
Ownership of practice setting					<.01			<.01
Private	18 729 (86.1)	10.6	9420 (94.2)	13.8		9309 (79.3)	7.2	
Public	3015 (13.9)	4.1	585 (5.8)	9.1		2430 (20.7)	2.9	
Accreditation level of practice setting					.35			<.01
Medical centre	3398 (15.6)	6.9	763 (7.6)	14.8		2635 (22.4)	4.6	
Regional/district hospital	5509 (25.3)	7.2	1525 (15.2)	14.2		3984 (33.9)	4.5	
Clinics	12 837 (59.0)	11.5	7717 (77.1)	13.3		5120 (43.6)	8.7	
Urbanization of practice					.62			.02
Urban	17 880 (82.2)	9.3	7724 (77.2)	13.5		10 156 (86.5)	6.1	
Non-urban	3864 (17.8)	11.3	2281 (22.8)	13.9		1583 (13.5)	7.7	

TWD30 ≌ 1USD.

**Table 4.** Patient and physician characteristics associated with antibiotic prescribing for dengue patients in outpatient and inpatient settings, Taiwan, 2008–15.

	Outpatient setting						Inpatient setting					
	Total		Pre-dengue confirmation		Post-dengue confirmation		Total		Pre-dengue confirmation		Post-dengue confirmation	
	AOR	(95% CI)	AOR	(95% CI)	AOR	(95% CI)	AOR	(95% CI)	AOR	(95% CI)	AOR	(95% CI)
Epidemic period												
No	ref		ref		ref		ref		ref		ref	
Yes	0.74	(0.63–0.88)	0.86	(0.72–1.04)	0.75	(0.54–1.04)	0.51	(0.39–0.67)	0.91	(0.60–1.38)	0.49	(0.33–0.71)
Patient characteristics												
Child age (years)												
<1	ref		ref		ref		ref		ref		ref	
1–4	1.86	(1.08–3.19)	2.70	(1.42–5.13)	1.30	(0.61–2.78)	0.56	(0.32–0.98)	0.61	(0.19–1.95)	0.60	(0.28–1.27)
5–9	1.88	(1.11–3.17)	2.63	(1.41–4.92)	1.50	(0.71–3.17)	0.44	(0.27–0.72)	0.69	(0.22–2.13)	0.40	(0.21–0.75)
10–14	2.07	(1.23–3.48)	3.51	(1.88–6.54)	1.35	(0.66–2.75)	0.24	(0.15–0.39)	0.36	(0.13–1.01)	0.24	(0.13–0.46)
15–17	2.59	(1.53–4.40)	4.88	(2.60–9.16)	1.56	(0.75–3.21)	0.28	(0.16–0.48)	0.44	(0.15–1.30)	0.28	(0.14–0.54)
Child sex												
Male	ref		ref		ref		ref		ref		ref	
Female	1.05	(0.96–1.1)	1.01	(0.90–1.14)	1.12	(0.96–1.32)	1.33	(1.10–1.59)	1.23	(0.87–1.75)	1.40	(1.11–1.78)
Parental SES												
<TWD30 000	ref		ref		ref		ref		ref		ref	
≥TWD30 000	1.01	(0.90–1.14)	1.04	(0.90–1.20)	0.97	(0.79–1.19)	1.05	(0.84–1.31)	1.39	(0.99–1.97)	1.04	(0.81–1.34)
Others	1.03	(0.91–1.16)	1.06	(0.92–1.23)	0.96	(0.78–1.19)	1.00	(0.80–1.25)	0.89	(0.58–1.36)	1.08	(0.80–1.47)
Child major comorbid conditions												
No	ref		ref		ref		ref		ref		ref	
Yes	1.17	(0.84–1.64)	1.22	(0.84–1.76)	1.06	(0.58–1.94)	1.88	(1.11–3.18)	1.79	(0.75–4.25)	1.71	(0.84–3.46)
Physician characteristics												
Physician age (years)												
<35	ref		ref		ref		ref		ref		ref	
36–59	1.12	(0.85–1.49)	1.13	(0.78–1.64)	1.09	(0.74–1.62)	1.48	(0.87–2.52)	1.42	(0.59–3.41)	1.57	(0.73–3.41)
≥60	1.78	(1.26–2.50)	1.65	(1.07–2.53)	1.98	(1.21–3.26)	0.65	(0.27–1.57)	0.45	(0.13–1.53)	0.84	(0.27–2.60)
Physician sex												
Male	ref		ref		ref		ref		ref		ref	
Female	1.00	(0.80–1.23)	1.07	(0.82–1.40)	1.26	(0.95–1.68)	1.16	(0.81–1.67)	1.23	(0.72–2.12)	1.35	(0.90–2.02)
Physician specialty												
Family medicine	ref		ref		ref		NE		NE		NE	
Internal medicine	0.94	(0.70–1.27)	0.90	(0.64–1.27)	1.30	(0.81–2.09)	NE		NE		NE	
Otolaryngology	2.87	(2.22–3.70)	2.48	(1.87–3.29)	4.17	(2.69–6.49)	NE		NE		NE	
Emergency	1.36	(0.97–1.91)	0.99	(0.64–1.51)	1.74	(1.04–2.93)	NE		NE		NE	
Paediatrics	1.19	(0.92–1.54)	1.14	(0.85–1.53)	1.70	(1.11–2.61)	ref		ref		ref	
Others	1.76	(1.29–2.41)	1.20	(0.81–1.79)	3.63	(2.21–5.95)	0.70	(0.43–1.13)	0.52	(0.26–1.02)	0.67	(0.39–1.15)
Average monthly volume												
Low	ref		ref		ref		ref		ref		ref	
Medium	1.17	(0.91–1.50)	1.04	(0.77–1.40)	1.23	(0.85–1.79)	1.11	(0.79–1.56)	1.03	(0.61–1.74)	1.11	(0.76–1.63)
High	1.03	(0.77–1.37)	0.96	(0.69–1.33)	1.08	(0.68–1.70)	1.44	(0.98–2.13)	1.01	(0.59–1.73)	1.72	(1.09–2.71)
Ownership of practice setting												
Private	ref		ref		ref		ref		ref		ref	
Public	0.78	(0.58–1.05)	0.88	(0.59–1.30)	0.76	(0.51–1.11)	0.78	(0.53–1.14)	0.54	(0.29–0.98)	0.91	(0.58–1.41)
Accreditation level of practice setting												
Medical centre	ref		ref		ref		ref		ref		ref	
Regional/district hospital	1.34	(1.00–1.79)	1.27	(0.85–1.88)	1.18	(0.81–1.71)	1.66	(1.10–2.52)	3.22	(1.74–5.94)	1.57	(1.00–2.46)
Clinics	1.46	(1.04–2.06)	0.88	(0.56–1.38)	1.30	(0.82–2.08)						
Urbanization of practice												
Urban	ref		ref		ref		ref		ref		ref	
Non-urban	1.26	(1.07–1.50)	1.20	(0.99–1.46)	1.28	(0.98–1.67)	0.92	(0.56–1.54)	0.54	(0.27–1.06)	0.94	(0.53–1.66)

*Note*: In inpatient settings analyses, physician specialty only categorized into paediatric and others, and accreditation level of practice setting only included medical centre and regional/district hospital. TWD30 ≌ 1USD. AOR is an odds ratio adjusting for all other independent variables in the model.AOR, adjusted odds ratio; NE, not evaluated; ref, reference.

Finally, the proportion of outpatient visits with antibiotic prescriptions decreased from 13.5% to 6.3% after dengue infection was confirmed. Prescription patterns across patient and physician characteristics were similar during the pre- and post-dengue confirmation weeks.

#### Antibiotic prescription in the inpatient setting

In this study, 25.1% of hospitalized children with dengue were prescribed antibiotics during the 14-day assessment period (Table 5). Antibiotics were prescribed less often during the epidemic years (23.6%) than in other years (37.2%)—(OR: 0.51; 95% CI: 0.39–0.67). In all, 38.8% of infants received antibiotics, compared to 20% of 10- to 14-year-old children. After adjustment, a clear dose–response relationship was observed between children’s age and antibiotic prescription during hospitalization; specifically, older children had a lower likelihood of receiving antibiotics. Children who were girls (OR: 1.33; 95% CI: 1.10–1.59) with any major comorbidities (OR: 1.88; 95% CI: 1.11–3.18) were more likely to be prescribed antibiotics during hospitalization. Physicians aged ≥36 years (36–59 years: OR: 0.24; 95% CI: 0.15–0.39; ≥60 years: OR: 0.28; 95% CI: 0.16–0.48) were significantly less likely to prescribe antibiotics in the inpatient setting. Physicians practicing in regional or district hospitals (OR: 1.10; 95% CI: 1.00–2.52) were more likely to prescribe antibiotics to hospitalized children with dengue.

**Table 5.** Inpatient antibiotic use for confirmed dengue by patient and physician characteristics, in pre- and post-dengue diagnosis periods, Taiwan, 2008–15.

	Total		Pre-dengue confirmation			Post-dengue confirmation		
		Antibiotics use		Antibiotics use			Antibiotics use	
	*N* (%)	%	*N* (%)	%	*P*	*N* (%)	%	*P*
Total admissions	2520	25.1	614	43.2		1906	19.3	
Epidemic period					.39			<.01
No	277 (11.0)	37.2	111 (18.1)	46.8		166 (8.7)	30.7	
Yes	2243 (89.0)	23.6	503 (81.9)	42.3		1740 (91.3)	18.2	
Patient characteristics								
Child age (years)					.03			<.01
<1	67 (2.7)	38.8	22 (3.6)	50.0		45 (2.4)	33.3	
1–4	231 (9.2)	30.7	73 (11.9)	43.8		158 (8.3)	24.7	
5–9	620 (24.6)	30.2	174 (28.3)	51.1		446 (23.4)	22.0	
10–14	931 (36.9)	20.0	205 (33.4)	34.6		726 (38.1)	15.8	
15–17	671 (26.6)	24.1	140 (22.8)	44.3		531 (27.9)	18.8	
Child sex					.59			<.01
Male	1508 (59.8)	23.2	369 (60.1)	42.3		1139 (59.8)	17.0	
Female	1012 (40.2)	27.9	245 (39.9)	44.5		767 (40.2)	22.6	
Parental SES					.27			.98
<TWD30 000	1120 (44.4)	25.1	293 (47.7)	41.3		827 (43.4)	19.3	
≥TWD30 000	737 (29.2)	25.9	165 (26.9)	48.5		572 (30.0)	19.4	
Others	663 (26.3)	24.1	156 (25.4)	41.0		507 (26.6)	18.9	
Child major comorbid conditions					.98			.66
No	2458 (97.5)	25.0	593 (96.6)	43.2		1865 (97.8)	19.2	
Yes	62 (2.5)	29.0	21 (3.4)	42.9		41 (2.2)	22.0	
Physician characteristics								
Physician age (years)					<.01			.03
<35	136 (5.4)	17.6	25 (4.1)	32.0		111 (5.8)	14.4	
36–59	2258 (89.6)	26.3	559 (91.0)	45.1		1699 (89.1)	20.1	
≥60	126 (5.0)	11.9	30 (4.9)	16.7		96 (5.0)	10.4	
Physician sex					<.01			<.01
Male	1744 (69.2)	22.3	423 (68.9)	38.8		1321 (69.3)	17.0	
Female	776 (30.8)	31.3	191 (31.1)	52.9		585 (30.7)	24.3	
Physician specialty					<.01			<.01
Paediatrics	2211 (87.7)	23.8	550 (89.6)	41.1		1661 (87.1)	18.1	
Others	309 (12.3)	34.3	64 (10.4)	60.9		245 (12.9)	27.3	
Average monthly volume					.07			<.01
Low	820 (32.5)	20.4	152 (24.8)	40.1		668 (35.0)	15.9	
Medium	835 (33.1)	18.7	184 (30.0)	38.0		651 (34.2)	13.2	
High	865 (34.3)	35.7	278 (45.3)	48.2		587 (30.8)	29.8	
Ownership of practice setting					.19			.00
Private	1725 (68.5)	27.5	466 (75.9)	44.6		1259 (66.1)	21.2	
Public	795 (31.5)	19.7	148 (24.1)	38.5		647 (33.9)	15.5	
Accreditation level of practice setting					<.01			<.01
Medical centre	1062 (42.1)	15.0	218 (35.5)	27.5		844 (44.3)	11.7	
Regional/district hospital	1458 (57.9)	32.4	396 (64.5)	51.8		1062 (55.7)	25.2	
Urbanization of practice					.96			.15
Urban	2161 (85.8)	24.0	464 (75.6)	43.1		1697 (89.0)	18.8	
Non-urban	359 (14.2)	31.5	150 (24.4)	43.3		209 (11.0)	23.0	

TWD30 ≌ 1USD.

Finally, the proportion of children prescribed antibiotics was substantially lower among children hospitalized after dengue infection confirmation (19.3%) than among children hospitalized before dengue infection confirmation (43.2%). A similar prescription pattern was observed for physicians prescribing antibiotics to hospitalized children with dengue after confirmation. In contrast, the prescription pattern in the preconfirmation period did not vary significantly across child or physician characteristics, except in relation to practice settings (Table 4).

## Discussion

### Statement of principal findings

This population-based study is one of the largest-scale assessment of antibiotic prescription patterns and related factors in children with confirmed dengue infection. As the study employed data from the Taiwan NHI scheme covering 99.9% of the island’s population, and because antibiotics should be available only by prescription, results amount to a near complete ascertainment of antibiotic use in confirmed dengue cases in Taiwan. By excluding children who had a concomitant bacterial infection during the 14-day assessment period around the date of laboratory confirmation, the observation that 29.4% of paediatric dengue cases received a course of antibiotics (23.4% of outpatient cases and 40.4% of hospitalized cases) is an accurate, if conservative, estimate of antibiotic use for dengue that potentially could be avoided in Taiwan if dengue was prevented. Moreover, in many dengue-endemic low- and middle-income countries, antibiotics are readily available without prescription and so potentially avoidable consumption would be even greater.

### Strengths and limitations

Several limitations of this study should be noted. Firstly, although the use of surveillance and claims data eliminated some of the shortcomings of survey data, certain inherent limitations of such data—including limited information regarding clinical presentation, parental SES, disease severity, and physician–patient communication—may have introduced confounding bias and hindered the detailed investigation of the factors influencing physicians’ prescription decisions. Secondly, because antibiotics that are paid out of pocket by parents, as the NHI may not cover them, are not recorded by the NHIRD, we may have underestimated the prevalence of antibiotic prescription in this study. Thirdly, although we excluded records with concomitant bacterial infection, misclassification bias may still exist. Finally, the present findings from Taiwan may not be generalizable to other settings with low insurance coverage or low access to outpatient care and inpatient services. Use of leftover antibiotics from previous encounters and provision by friends and family also cannot be ruled out.

### Interpretation within the context of the wider literature

This study uncovered some major findings. First, ∼30% of children with dengue were prescribed antibiotics during their medical visits within the 14-day assessment period. Antibiotic prescription for children with dengue was more prevalent in the inpatient setting than in the outpatient setting. Notably, in both settings, antibiotic prescription decreased to less than half after dengue was confirmed; this finding indicated that the non-specific symptoms or signs of dengue plays a significant role in physicians’ decisions to prescribe antibiotics. Our findings are consistent with previous studies in adults with dengue or acute febrile illnesses, demonstrating that a positive dengue test result can significantly reduce a patient’s likelihood of inappropriately receiving antibiotics in either the outpatient or inpatient setting [20, 21].

The frequent prescription of antibiotics in hospitalized children with dengue is a major concern. In this study, ∼20% of hospitalized children with dengue received antibiotics after dengue had been confirmed, even though we had excluded all records with concomitant bacterial infection diagnosis from our analyses. A similar observation was made in a study of children with leucocytosis who were treated for other viral infections in Taiwan [22]. In our analyses, we identified high-risk groups of hospitalized children with dengue receiving inappropriate antibiotics, with higher antibiotic prescription observed in infants and children aged <5 years as well as in girls. Contrasting associations were found between child age and antibiotic prescription in outpatient and inpatient settings; specifically, physicians were more likely to prescribe antibiotics to young children in the inpatient setting than in the outpatient setting. One plausible explanation for this outcome is that the progression of dengue in hospitalized younger children tends to be more severe, less certain, and associated with higher risk; thus, antibiotics may be prescribed as a precaution. Owing to data limitations, we were unable to identify clinical symptoms or dengue severity. In this regard, future research could assess the influence of dengue severity and the clinical symptoms of hospitalized children on antibiotic prescription decisions. In the outpatient setting, school-age children are more likely to be prescribed antibiotics than are their younger counterparts. This may be because under time constraints, physicians may not be able to conduct comprehensive assessments. Hence, as a precaution, they might prescribe antibiotics to reduce the likelihood of return visits and of school absences for school-age children.

Physician characteristics played a significant role in antibiotic prescription for children with dengue, particularly in the outpatient setting. The rate of antibiotic prescription for children with dengue in the outpatient setting was not as high as that of antibiotic prescription for acute viral respiratory illnesses such as tonsillitis in Taiwan [12, 23]. Consistent with prior studies, significant variations were observed across physicians’ age and specialty in the outpatient setting, likely because of variations in medical education or training in medical schools or residency programmes. In the medical school curriculum and in residency programmes, training regarding the cautious prescription of antibiotics should be provided to cultivate more appropriate prescription habits in doctors-in training. In addition, consistent with prior studies [12, 24], the practice setting of physicians independently contributed to the antibiotic prescription decisions. Specifically, physicians practicing in lower-level hospitals or clinics or in rural areas appeared to be more inclined to prescribe antibiotics than their counterparts; this observation was likely due to the physicians’ relatively limited access to laboratory and testing facilities, as well as their experiences of less peer pressure and less demand for compliance with up-to-date guidelines, compared with their counterparts working in medical centres or in urban areas [25, 26].

Consistent with prior studies focusing on adults [27–29], in the present study, antibiotics were prescribed less often in the epidemic period than in usual years. While dengue is introduced frequently to Taiwan, when local outbreaks occur, they are usually limited in size and geographic range. A series of outbreaks in 2014–2015 was exceptional, leading to case numbers two orders of magnitude greater than in usual years. Because of its frequently severe but non-specific clinical manifestations, dengue is commonly treated with antibiotics. However, during the epidemic period, more intensive dissemination of dengue-related information and stronger dengue control efforts may have led to heightened awareness of the diagnosis among physicians, leading them to consider dengue as the likely cause of febrile illnesses during this interval and to modify their prescribing behaviour.

### Implications for policy, practice, and research

Several approaches could effectively strengthen antibiotic stewardship, such as increasing the availability of rapid, reliable, and sensitive dengue diagnostic tools at the point of care [30], improving access to laboratory and testing facilities, providing educational training, and offering financial incentives to such physicians for the cautious prescription of antibiotics. Furthermore, the time constraints and a high patient load could substantially compromise a physician’s cautious prescription of antibiotics. Therefore, physicians’ working conditions should be considered when examining the factors influencing their prescription behaviours. Control and prevention of dengue itself, by improved vector management and immunization, to reduce or prevent cases from occurring in the first place, should decrease the occasions when antibiotics may be used inappropriately.

## Conclusions

This population-based study examined the complex interplay of factors influencing antibiotic prescription including a wide range of child, parent, and health care provider characteristics in both outpatient and inpatient settings. We observed the continued use of antibiotics for dengue in Taiwanese children even after the diagnosis was confirmed. Mitigation should target modifiable characteristics including the use of rapid point-of-care tests and timely access to results of confirmatory diagnosis and site-specific antibiotic stewardship approaches based on the identified health care delivery and provider characteristics.

## Supplementary Material

mzae052_Supp

## Data Availability

This work is based on data from the Health and Welfare Data Science Center, Ministry of Health and Welfare (HWDC, MOHW), Taiwan (contact via https://dep.mohw.gov.tw/dos/cp-5119-59201-113.html). All these databases are available for research purposes, managed by the HWDC, and linked using encrypted identification numbers of individual patients, physicians, and hospitals. All the analyses were limited to be conducted onsite at the HWDC.
